# The Evolving Role of Microsampling in Therapeutic Drug Monitoring of Monoclonal Antibodies in Inflammatory Diseases

**DOI:** 10.3390/molecules26061787

**Published:** 2021-03-22

**Authors:** Panagiotis-Dimitrios Mingas, Jurij Zdovc, Iztok Grabnar, Tomaž Vovk

**Affiliations:** Chair of Biopharmaceutics and Pharmacokinetics, Faculty of Pharmacy, University of Ljubljana, Aškerčeva 7, 1000 Ljubljana, Slovenia; Panagiotis-Dimitrios.mingas@ffa.uni-lj.si (P.-D.M.); Jurij.Zdovc@ffa.uni-lj.si (J.Z.); Iztok.Grabnar@ffa.uni-lj.si (I.G.)

**Keywords:** microsampling, DBS, VAMS, mAbs, TDM, inflammatory diseases

## Abstract

Monoclonal antibodies (mAbs) have been extensively developed over the past few years, for the treatment of various inflammatory diseases. They are large molecules characterized by complex pharmacokinetic and pharmacodynamic properties. Therapeutic drug monitoring (TDM) is routinely implemented in the therapy with mAbs, to monitor patients’ treatment response and to further guide dose adjustments. Serum has been the matrix of choice in the TDM of mAbs and its sampling requires the visit of the patients to laboratories that are not always easily accessible. Therefore, dried blood spots (DBS) and various microsampling techniques have been suggested as an alternative. DBS is a sampling technique in which capillary blood is deposited on a special filter paper. It is a relatively simple procedure, and the patients can perform the home-sampling. The convenience it offers has enabled its use in the quantification of small-molecule drugs, whilst in the recent years, studies aimed to develop microsampling methods that will facilitate the TDM of mAbs. Nevertheless, hematocrit still remains an obstacle that hinders a more widespread implementation of DBS in clinical practice. The introduction of novel analytical techniques and contemporary microsampling devices can be considered the steppingstone to the attempts made addressing this issue.

## 1. Introduction

During the past three decades there has been a transition in the type of therapeutics in drug development. The focus has shifted towards large-molecule drugs, such as proteins and peptides. The most prevailing class of drugs under development are the monoclonal antibodies (mAbs) which have been extensively developed for various inflammatory diseases (e.g., inflammatory bowel disease (IBD), atopic dermatitis, psoriasis, rheumatic diseases). Antibodies are large proteins (∼150 kDa), also known as immunoglobulins, utilized by the immune system for the detection and further elimination of specific antigens, such as bacteria and viruses [1,2]. In 1975, Köhler and Milstein [3] developed the hybridoma technique, an important step for deriving mAbs in large amounts and thus improving the research and possibility for further clinical use. Currently, 79 therapeutic mAbs have already been approved by the United States Food and Drug Administration (US FDA); meanwhile at least 570 mAbs are in different developmental stages [4]. In general, mAbs are characterized by complex pharmacokinetics and pharmacodynamics when compared to non-antibody-type drugs [5]. They hold multiple favorable pharmacological characteristics such as extended serum half-life, increased specificity and as a result lower risk for off-target toxicity [2]. Properties such as susceptibility to oral degradation, molecular size, hydrophilicity, and poor membrane permeability are among the causes for a predominant parenteral administration of the therapeutic proteins mostly by intravenous infusion, but either subcutaneous or intramuscular injection are possible [5,6].

The mAbs can be found in serum in either free, unbound form, or in a complex, formed with the target antigen or the anti-drug antibodies (ADAs). ADAs are endogenous antibodies and represent the immune response against exogenous mAbs. Overall, humanized or fully human mAbs induce an immune response less often than murine antibodies. The mAb–ADAs complex is generally inactive; increased number of ADAs can lead to less efficacious treatment because of the decreased number of free mAbs able to bind to the target antigen, in order to exert their pharmacologic action. As a result, patients who responded well to the treatment with therapeutic mAbs, might gradually lose response to the therapy, partly because of the increased presence of ADAs. The underlying mechanisms can be rather complex and monitoring of the serum mAb concentration is considered important in order to further proceed with stratified dose adjustments or possible switch to another therapy [7,8]. Usually, this is done within the procedure of therapeutic drug monitoring (TDM) which is a multidisciplinary clinical specialty aiming to improve patient care. This approach can be based on either prior pharmacogenetic, demographic and clinical information, posterior measurement of drug concentration in blood (pharmacokinetic monitoring) and/or measurement of biomarkers, which can be either pharmacodynamic, i.e., monitoring of the biomarkers related with the drug effect; or disease-related, i.e., following the severity or presence of some disease state [9].

**Figure 1:**
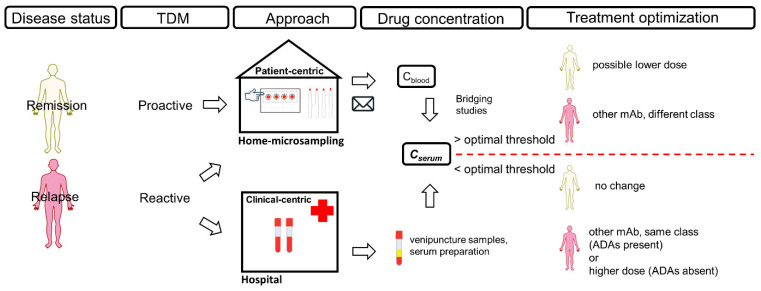
TDM process involving microsampling.

TDM is mainly performed by collecting venous blood samples in specific institutions or hospitals, which are not always easily accessible to every patient. In contrast, microsampling allows convenient home-sampling while requiring low volume of samples (≤50 μL) (Figure 1). The most traditional microsampling approach is dried blood spots (DBS), in which capillary blood is most often obtained after a finger prick on a special filter paper. The DBS could be sent to a laboratory by post, subsequently extracted and the analyte concentration is then determined using an appropriate analytical technique. Many analytes are sensitive to hydrolysis, yet their stability in DBS is often better preserved in comparison to frozen serum or plasma samples, offering an easy way of storage and transport. DBS is a relatively simple procedure, and the patients are usually capable to perform the home-sampling after comprehensible instructions, on how to avoid unsuccessful or contaminated samples [10,11,12,13]. The utilization of DBS has been mostly investigated for the quantification of small-molecule drugs in toxicokinetic, pharmacodynamic and pharmacokinetic (PK) studies [14,15]. During the coronavirus disease 2019 (COVID-19) pandemic, this technique becomes even more relevant. A recent development and validation of new kits that enable the collection of DBS samples at home, showed comparable analytical results to samples obtained after venipuncture. Such methods could greatly support the health care system, by decreasing the burden of serological testing [16].

The aim of this article is to present novel microsampling approaches that have been used in the TDM of mAbs in various inflammatory diseases. Additionally, strategies that have been developed to further assist the implementation of a patient-centric approach in the clinical practice will be discussed.

## 2. Therapeutic Drug Monitoring of Monoclonal Antibodies

In recent years, mAbs have contributed to better therapeutic success in management of inflammatory diseases [17,18]. Still, not all patients typically respond to therapy in the same way, and the TDM can be used as a tool for informed clinical decision making and dose adjustments, focused to maximize the response of patients [19,20]. In current practice, the concentration of mAbs is usually monitored in serum after the peripheral venipuncture. The sampling is mostly performed at trough (i.e., immediately before the next dose), since the trough serum concentration well reflects the overall exposure to the drug and systemic drug clearance, although monitoring at other time-points may be possible, as well. The serum concentration is subsequently compared to the established optimal drug concentration, at which the patient is most likely to respond, based on the known exposure-response relationship [21]. The optimal concentration is specific for each mAb, disease indication, dosing regimen and treatment time-point. To date, several reviews have summarized the studies assessing the exposure-response relationships and optimal concentration thresholds for mAbs in inflammatory diseases [22,23,24,25,26]. It seems the consensus and guidelines for TDM and target mAb concentrations are best established in the field of Gastroenterology, but the advances are continuously made in the management of rheumatic and dermatologic conditions, as well (Table 1) [23,27,28,29,30,31,32,33].

TDM can be done reactively, as a consequence to a patient’s nonresponse to treatment, or proactively, when the patient is in a state of remission and the main purpose of TDM is prophylactic [35,36]. In general, the lack or loss of response to treatment with mAbs is related with several mechanistic or non-mechanistic factors. If a nonresponding patient has optimal serum drug concentration, the reason is likely a mechanistic, disease-related failure, and occurs when the pathological immune response is driven by mediators unaffected by the drug. In this case, the patient is unlikely to respond to mAbs acting on the same target, and a switch to a different class mAb may be necessary. If a patient has suboptimal drug concentration, then ADAs should be considered, as well. The presence of ADAs indicates an immune-mediated nonresponse due to drug immunogenicity, which might be avoided by switching the therapy to another mAb from the same class. In contrast, the absence of ADAs together with suboptimal drug concentration indicate a non-immune mediated pharmacokinetic nonresponse, which might be overcome by dose adjustment, to achieve higher exposure and drug concentration, at which the patient is more likely to respond to treatment [19,23,26,27,28,37].

The variability in response is partly related with variable PK of mAbs. Commonly, therapeutic mAbs are IgG type of antibodies, and have distinct PK characteristics compared to smaller molecules. High molecular mass and hydrophilic properties result in poor permeability through tissue membranes, and usually limit the mAb distribution to plasma. In addition, mAbs are generally characterized with a slow systemic clearance and long terminal half-life. Their catabolism may be divided in two main pathways: a linear, non-specific clearance, which is predominantly mediated by the intracellular lysosomal degradation into amino acids; and a nonlinear, specific clearance, mediated by the binding of the antibody to its target [6,38,39]. The non-specific clearance is influenced by the protective mechanism for IgG molecules, provided by the Brambell receptor. This mechanism prolongs the half-life of IgG antibodies to around 20 days, which is significantly longer compared to other proteins with similar size and reduces the frequency of dosing. In contrast, nonlinear target-mediated clearance varies with different inflammation status, due to the variable concentration of the inflammation mediators, which interact with the mAb [6,38,39,40]. All of these factors eventually translate into a variable mAb concentration, which is monitored, and translated into the clinical action. Therefore, the assays for monitoring the mAb concentration should be accurate and precise, as it can otherwise result in an inappropriate therapeutic decision. Moreover, considering mAbs are frequently used in chronic diseases requiring treatment over longer period, microsampling methods could add important value to TDM by allowing frequent and convenient concentration monitoring, and timely dose adjustment, which would further maximize the patients’ response. Compared to traditional sampling procedure, microsampling seems better suited especially for the proactive TDM, even though the cost-effectiveness of the latter is still unclear [41,42,43].

## 3. Therapeutic Drug Monitoring of mAbs Utilizing a Patient-Centric Approach

Current trends of the TDM process involve patient-centric approaches utilizing easy and painless sampling, while ensuring high quality samples [44,45]. Microsampling is one of the key steps in this process since it allows possible home-sampling, by simple collection of the capillary blood which can be performed by the patient. The collection of the blood from the finger prick is generally accepted as less painful than phlebotomy and after appropriate education patients are able to prepare quality microsamples, that are comparable to those collected by clinical staff [46]. Nevertheless, the challenges of finger prick sampling may be associated with poor peripheral circulation, slow bleeding and in rare instances pain [44,47], thus forcing researchers to search for advanced sampling strategies. One of the new sampling devices e.g., Tasso OnDemand™ is designed for collection of the capillary blood from the upper arm, where capillary blood flow is better and sampling process is less painful than from fingers [44,48]. The collection of the capillary blood is either performed by non-volumetric (e.g., DBS) or by volumetric approaches (e.g., volumetric absorptive microsampling (VAMS)), since both types of microsampling enable preparation of high-quality samples by the patient. After the blood samples are collected, it is generally recommended (regardless of the type of a microsample), that they should be air dried at room temperature conditions for at least 1 to 3 h. Subsequently, they must be stored in a tightly closed container with a desiccant, to avoid degradation of the analytes due to natural humidity fluctuations, protected from the sun [9,49]. It is particularly important that during the development and validation of novel analytical methods, the influence of relative humidity, the quality of microsamples, the type of filter paper and the temperature (during storage and shipment of the microsamples) are evaluated. Moreover, the stability of the analytes should be examined in all conditions that are expected to be met during the different stages of the microsamples handling [9,50,51,52]. Validated microsampling methods can promote patient-centric approaches in TDM of mAbs by reducing hospital visits and consequently the possibility of infection exposure, while enabling the clinicians to maintain the adequate patient control and the optimal individualized posology [41,53,54,55,56].

Another obstacle in the implementation of the microsampling to the TDM process arises from the difference between capillary samples and traditional TDM samples e.g., serum or plasma derived from the venous blood. To confirm that concentration data obtained from the alternative microsampling approach is equivalent with traditional samples, bridging studies that overcome analytical and physiological issues need to be included into the validation process [9,57,58]. Small sample volume and complex matrix demand analytical method development to focus on extraction procedures and selection of sensitive analytical methods. The analyte partitioning between plasma and blood cells can significantly influence on the concentrations determined in plasma or blood samples. Nevertheless, mAbs are large proteins and thus partitioning into the blood cells is not expected. Therefore, inclusion of the fixed [53,54] or individual [41] hematocrit (Hct) values into the models that relate plasma and blood concentrations, may improve accuracy and precision. Bridging studies should also address the differences arising from venous blood sampling using anticoagulant and microsampling where anticoagulant is not included [9]. Nevertheless, studies developing microsampling methods for mAbs as an alternative for TDM are recently increasing, and a summary of these studies is presented (Table 2).

### 3.1. Tumor Necrosis Factor Inhibitors

#### 3.1.1. Adalimumab

A cross-sectional study was conducted [53], including 161 patients, diagnosed with rheumatic inflammatory diseases (rheumatoid arthritis, psoriatic arthritis and ankylosing spondylitis), and treated with adalimumab. The DBS samples (after finger prick) and serum samples (after venipuncture) were simultaneously obtained. The surface area of the DBS was first measured, and then the entire DBS was eluted. The extracts were preserved at 4 °C, until the concentration measurements for adalimumab and ADAs were carried out, by enzyme-linked immunosorbent assay (ELISA) and antigen-binding test, respectively. The concentrations in the DBS samples were converted to DBS-serum concentration by using the DBS H0.42 method, which utilizes a fixed Hct of 0.42 combined with the DBS area. The converted DBS-serum concentrations were then compared to serum samples concentrations. The converted DBS-serum drug concentration was lower over the range of analyzed concentrations, which indicated a discrepancy in the concentration of proteins in capillary blood in comparison to venous blood. To correct this bias, a constant factor of 1.19 was calculated, based on the median deviation of −16.26% in the adalimumab concentration in DBS-serum concentrations in comparison to the venipuncture serum samples. This study demonstrated that adalimumab concentration, as well as ADA concentration, could be adequately measured in DBS samples, obtained after finger prick.

A recent study [54] was conducted aiming to compare the adalimumab serum concentrations obtained after venipuncture to those collected via the volumetric method of VAMS. The study included 33 patients diagnosed with IBD. During their visit to the outpatient clinic, venous blood was obtained firstly via venipuncture followed by the collection of two capillary blood samples: the first obtained by the healthcare personnel and the second by the patients themselves, after appropriate education. Before their next visit at the clinic, the patients were also asked to obtain a VAMS sample at their home and send it to a laboratory. Both serum and capillary blood samples were measured by ELISA. The capillary blood samples were converted to serum adalimumab concentrations, by using a fixed Hct value of 0.42. The results showed a high correlation between capillary blood, obtained by healthcare professionals and patients at the clinic, and venipuncture samples (r = 0.96 and r = 0.97, respectively). The capillary blood samples obtained by the patients at home were compared to predicted, by using Bayesian analysis, adalimumab serum concentrations. There was observed neither proportional nor systemic bias, but high variability was demonstrated between home-sampling VAMS and predicted serum adalimumab concentrations (accuracy 45%). In conclusion, the high agreement observed between the converted capillary blood and serum concentrations, suggested that the use of microsampling in clinical practice could potentially shorten the time intervals needed for adalimumab dose adjustments, as well as possibly lead to improved treatment outcomes.

#### 3.1.2. Infliximab

Approximately 20 years ago, infliximab was the first approved anti-TNF mAb for the treatment of moderate to severe IBD. So far, all available data demonstrate that infliximab is very effective for the treatment of IBD. Nevertheless, there are concerns over its optimal dosing regimen and the possible formation of ADA [43,59]. Berends et al. [41], aimed to further optimize infliximab efficacy in patients diagnosed with IBD, by developing a method that could enable the home sampling, shortening the time until the next dose interval adjustment. In this study, a total of 40 IBD patients were included. The infliximab concentrations in capillary blood samples (obtained by finger prick) were compared to serum samples (collected after venipuncture). The capillary blood samples at different time points were collected using Mitra^®^, a novel microsampling device with VAMS technology, either at the hospital by the assistance of experienced personnel or at home by the patients. The Hct values of each patient were used to convert the DBS extract concentrations to serum concentrations. An accurate estimation of the infliximab serum concentrations from the DBS extracts was achieved, by using the DBS H-Hb (hemoglobin) method. The correlation between DBS and serum concentrations was higher for the samples collected at the hospital, compared to those collected at home (r ≥ 0.965 vs. r ≥ 0.671, respectively). The study demonstrated that samples collected utilizing the VAMS, offer a convenient method for measurement of infliximab concentration in IBD patients and improves the TDM. Additionally, the study emphasized the importance of patient education in the TDM using patient-centric microsampling approach.

#### 3.1.3. Golimumab

Golimumab was the latest approved anti-TNF mAb for the treatment of moderate to severe ulcerative colitis (UC), following adalimumab and infliximab. It is considered a safe and effective treatment but its optimal dosing regimen, possible combination with other immunomodulators and the TDM implementation, are still under investigation [60]. Detrez et al. [55] focused on the development of a DBS method for the determination of golimumab concentration, to provide a better understanding of the total drug exposure. Subsequently, the developed method was applied on DBS samples obtained from ten patients (included in GOUDA study) diagnosed with UC and treated with golimumab. Samples were collected by either venipuncture (serum) or finger pricking (DBS). The patients were educated on how to execute the home sampling and they positively approached the procedure. The DBS golimumab concentrations were converted to DBS-serum concentrations by using a mean conversion factor of 3.9, which combined the extraction recovery and the capillary blood/serum ratio of golimumab. The researchers concluded that the golimumab concentrations obtained after analysis of serum and DBS samples after their conversion to DBS-serum concentrations, simultaneously collected, correlated well (r = 0.990, *p* < 0.0001). The observed Hct of the patients was within the normal range (from 0.39 to 0.48) and therefore, the influence of Hct on golimumab concentrations was considered to be low. As such, the DBS contributed to the simplification of the TDM and offered a better understanding of the golimumab pharmacokinetics.

### 3.2. Monoclonal Antibodies with Various Mechanism of Actions

#### 3.2.1. Vedolizumab

Vedolizumab is a humanized mAb, characterized by a different mechanism of action and specifically acts on α4β7 integrin. The specific integrin is a therapeutic target for IBD, and vedolizumab is indicated for the treatment of patients who have been diagnosed with moderate to severe UC or Crohn’s disease (CD) [61,62]. Bian et al. [56] developed and validated a DBS method for the determination of vedolizumab concentrations in IBD patients. The patients’ DBS and serum samples were obtained by finger prick and venipuncture respectively at the same time, during the routine hospital visit. Following the measurement of the concentration for every sample, an individual vedolizumab DBS/serum concentration ratio was also calculated, for every patient. The DBS vedolizumab concentration ([VDZ]_DBS_) strongly correlated with the serum concentration ([VDZ]_serum_) (r = 0.978, *p* < 0.0001, n = 40) and was expressed by a linear regression: [VDZ]_DBS_ = [VDZ]_serum_ × 0.435 + 0.995 (R^2^ = 0.956, *p* < 0.0001). The results of the study suggested that the DBS method could be further utilized for the patient-centric TDM of vedolizumab.

#### 3.2.2. Others

Bloem et al. [63] took into consideration the challenges hindering a more extensive use of DBS, while developing methods for determining the concentration of numerous mAbs. These issues are related with the influence of Hct on the approximation of the blood volume of a DBS, which will be further discussed in the next chapters. Additionally, the distribution of mAbs is mostly limited to plasma. Therefore, a serum/plasma concentration should be defined. These concerns were individually analyzed, as part of the measurement of the concentration of those mAbs, namely adalimumab, infliximab, ustekinumab, vedolizumab, tocilizumab, natalizumab and rituximab. Except for the traditional DBS microsampling using Whatman^®^ filter paper, the Mitra^®^ microsampler that utilizes the VAMS principle, was also tested. The recovery for the Mitra^®^ microsampler was 95.2%, whereas for the Whatman^®^ paper 92.9% (standard deviations of 10.2 and 11.7 respectively). As a conclusion, this new type of microsampling was proven to be a user-friendly alternative to traditional DBS, for the determination of serum concentrations of various monoclonal antibodies, since the Mitra^®^ microsampler demonstrated a moderately lower variation of the obtained samples, in comparison to traditional DBS.

More specifically, ustekinumab (similarly to vedolizumab) is an alternative mAb for the treatment of IBD. It has demonstrated very good effectiveness in CD patients, who previously failed treatment with anti-TNF [64]. Recent studies have indicated that serum concentrations of ustekinumab in patients with CD, following an hour after intravenous infusion, can be utilized for the further optimization of the treatment [65]. Van den Berghe et al. [66], intended to develop and subsequently validate a DBS method, for the identification of the most appropriate time points to determine the ustekinumab concentrations, for the prediction of the treatment outcome, as well as for the investigation of its PK profile. The correlation of the ustekinumab in DBS extracts and serum samples was assessed by simultaneously collecting samples from 8 patients at two different time points. The concentrations of ustekinumab in DBS extracts (range: 0.55–12.1 μg/mL) correlated very well with those in serum (r = 0.982, *p* < 0.0001). Nevertheless, the investigation of the PK profile of ustekinumab and the determination of the best time points during induction for the prediction of treatment outcomes, would require the collection of more DBS samples.

## 4. Strategies for Correcting the Limitations of DBS

Despite the benefits DBS offers in sample collection, the assay bias caused by the Hct still remains an issue for the quantitative DBS analysis, impeding its more widespread implementation in clinical practice. Hematocrit is defined as the ratio of the volume of red blood cells to the total volume of the blood, and its most evident effect is on blood viscosity [67,68,69]. The Hct-based bias consists of: Hct-based recovery bias, Hct-based area bias and Hct-based matrix effect bias. It was demonstrated that the influence of the Hct-based recovery bias increases when the absolute recovery of the DBS assays decreases. Accordingly, the assays with high recoveries (over 90%) are not importantly influenced by the Hct-based recovery bias. On the contrary, the Hct-based recovery bias was more pronounced in assays with lower recoveries [70,71]. For the Hct-based recovery bias, internal standard (IS) application approaches can be used and the ability for co-extraction of analyte and IS for the elimination of the specific bias may be accounted [70]. The Hct-based area bias has been extensively discussed and is the result of variable spreading and homogeneity of the blood on the cellulose filter paper [67,68,69]. Chao et al. [72] demonstrated that the size and the kinetics of a drop of blood on Whatman^®^ 903 filter paper are proportionally reduced, with higher levels of Hct observed in blood. As an example, a blood sample that has been obtained from a patient with a high Hct (0.5) will spread less on a cellulose DBS card, compared to a blood sample obtained from a patient with a low Hct (0.3) [67]. The third type of bias, Hct-based matrix effect bias, originates from the fact that DBS samples with different Hct may be considered as different matrices. The matrix effect bias can influence the accuracy of the quantitative analysis, causing either under- or over-estimation of the results. Hence, during the development and validation of a method, the effect of Hct on these parameters should be considered. Several methods have been developed to overcome these issues [67].

### 4.1. Determination of Blood Volume in DBS Samples

The first step towards the correction of the Hct-based area bias which can lead to improved accuracy of the analytical results can be the determination of the volume of blood on a DBS or a DBS sub-punch, which can be performed even when the Hct value has not been determined. The rationale behind such approach is that a measured analyte concentration should be the same, whether it is measured in an equal volume of dried or liquid blood. A disadvantage of these methods is that while they enable the correction of the Hct-based area bias, they do not account for the effect the Hct-based recovery and Hct-based matrix effect biases can have on a quantitative measurement [67].

#### 4.1.1. Electrical Conductivity Measurement

Kadjo et al. [73], suggested a nondestructive method for the estimation of the volume of blood on a DBS, by measuring the electrical conductivity of a DBS extract. Prior to analysis, a DBS punch was obtained (diameter of 3–3.2 mm) and then extracted in water or water/methanol (or ethanol) mixtures. The principle of this method was based on the relatively constant electrolyte concentration in blood. Namely, the concentration of one of the most prevalent ions in blood, Na+, lies within the range of 120–150 mM, which was demonstrated in the 99.5% of over 111,000 analyzed blood samples. However, even though Na+ concentration in blood is higher compared to other ions, Cl− has 50% higher mobility and contributes most to the conductivity measurement. When calibrated in an appropriate range of electrolyte concentration, the electrical conductivity measurement of the DBS extract could estimate the blood volume in the DBS punch. The presented data were related to healthy individuals. This method might be hindered by possible abnormal electrolyte concentration, encountered in specific diseases.

#### 4.1.2. Capillary Electrophoresis with Capacitively Coupled Contactless Conductivity Detection

An approach based on a similar principle was applied by Dvořák et al. [74]. Their all-in-one approach simultaneously enabled the estimation of the volume of the blood on a DBS and the quantification of the analyte. Three extraction solvents were tested: deionized (DI) water, 50% (*v/v*) methanol/DI water and 100% methanol. The conductivities of each solvent were evaluated with the method of capacitively coupled contactless conductivity detection (CE-C4D), and the corresponding curves demonstrated very good linearity (R^2^ ≥ 0.9971), characterized by different slope and intercept values. In the next step, the DBS extracts were directly submitted for an automated analysis by CE-C4D and the concentrations of the inorganic contents of the blood (K+, Na+, Cl−) were estimated, after analyzing the response with a set of standard NaCl solutions. The results showed a strong linear relationship (R^2^ ≥ 0.9926) between the volume on the DBS and the inorganic ions contents, in the expected conductivity range. The stability studies of the DBS prepared one month in advance was also investigated, and no variation in the conductivity measurements was observed. The developed method was applied for the quantification of amino acids in DBS with unknown volume. The peak areas of K+ and Na+ were used to estimate the volume of the capillary blood on the DBS sub-punch which was estimated as 19.4 ± 0.8 μL. As such, this method could be useful in the case of a DBS with an unknown blood volume. The results that were obtained by either cutting the whole DBS or by a sub-punch of a small part of a DBS, were very good with variations between true and determined volume being ≤ 5.5%. Therefore, CE-C4D analysis could be used for the estimation of the volume of blood on a DBS, by quantifying inorganic constituents and subsequently for the quantification of specific analytes.

#### 4.1.3. Mathematical and Computational Approaches for the Correction of Hct Effect

A study by Alsous et al. [75] investigated the correlation between volume, Hct and surface area of DBS. A model relating all three parameters was developed, allowing the estimation of one parameter, based on the known values of the other two. For the method development, blood from healthy volunteers was obtained. Pre-determined amount of red blood cells and plasma were mixed in order to receive blood with variable Hct (0.25, 0.3, 0.35, 0.4, 0.45, 0.5 and 0.55). This range covered the anticipated Hct levels in patients. In the next step, DBS with various volumes (7.5, 10, 12.5, 15, 20, 25, and 30 μL) were spotted on Whatman^®^ 903 cards and subsequently dried. The DBS cards were then scanned, and the surface area was determined by utilizing the image processing program, ImageJ^®^. The dependent variable was the surface area, whereas Hct and volume of blood were the independent variables for the regression analysis. An external validation of the model was also performed, by spotting different Hct levels and volumes of blood within the range that was determined during the model development. The final model obtained was:

SA = (690.414 × BV) − (72.3 × HCT%) + 3941.8(1)

where SA is the surface area, BV the blood volume (μL) and Hct the hematocrit. During the validation, the model predicted the surface area with high precision (r = 0.999) and low bias (−6.09 and 9.07%). The experiments were performed using blank blood. It would be useful, if the influence of drugs (with various physicochemical properties) and their effect on spreading of the DBS on a card, was further assessed.

The effect of Hct on DBS was evaluated in a computational study by Daousani et al. [76]. The aim of the research was the suggestion of a Hct effect correction, which could be further implemented in DBS quantitative analysis, in case a DBS partial spot is analyzed. Therefore, special emphasis was given on the determination of a Hct range for adults, within which the correction of concentrations of unknown samples would not be necessary. This could be achieved by pre-determining an acceptable tolerance level for the Hct influence to the analytical total error. The result of the studies showed that it is crucial to establish strategies to assess the Hct effect in analysis of DBS in which a partial spot is obtained. It was demonstrated that preparing calibration standards and QC samples at a pre-determined Hct value, that was chosen based on demographic data is justified and it can result in an acceptable tolerated percentage of relative error associated to Hct effect. This is important because lower volumes of blood are required for further analysis. An upper level of 3% relative error, was considered an acceptable influence of the Hct, to the percentage of the total analytical error. This percentage was recommended after the theoretical studies that were conducted, but it can be variable depending on different analytes.

### 4.2. Determination of Hct in DBS Samples

As previously mentioned, the Hct is one of the most discussed problems that occur in analysis of DBS and it is generally considered as one of the most crucial factors that hinder their extensive use. A strategy to overcome this issue, is to prepare quality control DBS sample during the validation experiments that cover a range of different Hct values. Additionally, calibrators are also prepared using blood with Hct value close to the average Hct of the target population. Despite that, even when the Hct interval has been determined in which the assay bias falls within acceptable limits (generally ± 15%), it has to be known if the DBS sample lies within the validated Hct range [77]. As a result, the alternative approach that has been suggested is to directly measure or estimate the Hct of a DBS, by utilizing newly developed techniques.

#### 4.2.1. Image Analysis

To conduct the image analysis of the DBS cards, Fiji software can be used. It is a distribution of the open-source software ImageJ, that has been developed for biological image analysis [78]. Del Ben et al. [79] developed a nondestructive method using Fiji. After the collection of DBS samples, the DBS cards were scanned. In each spot a circular region of interest (ROI) was taken in the center of each spot and the mean gray value (MGV) of each ROI was obtained; these values range from 0 (black) to 255 (white). A Deming regression model was applied on the results and the equation of the linear regression was:

MGV = −171.2 × Hct + 158.4(2)

The simplicity of this procedure, its nondestructive nature, the low computational requirements, and the small number of patients are the main advantages of this method.

#### 4.2.2. UV-Visible Reflectance and Near Infrared Spectroscopy

The first attempt to address the Hct issue, by trying to estimate the hemoglobin (Hb) was done by Miller IV et al. [80]. Ultra-violet (UV)–visible (VIS) reference measurements were carried out by taking ambient measurements, in which the source of light was blocked. They examined the correlation between the Hct of a DBS and the reflectance at 540 and/or 570 nm, two wavelengths that are specific for Hb, but no correlation was observed. However, an association was found between the background scattering at 980 nm and the Hct of the DBS. Therefore, it was possible to determine the sample volume in a DBS punch of 3 mm. This method was suggested for similarly sized DBS, because the volume of the blood on the DBS can affect the measured reflectance of spots with the same Hct. In addition, they recommended the use of this technique for DBS obtained from pre-printed filter paper, to enable the assessment of DBS size [67]. However, the effect of the age of the DBS on the measured reflectance was not evaluated during the validation. The intensity of the color on a DBS changes with time and can significantly affect the reflectance results [69].

Another similar spectroscopic approach, developed by Capiau et al. [81,82], was based on diffuse UV-VIS reflectance and it was used to estimate the Hct from a DBS, as well. In this technique, broadband light from a halogen source is directed to the surface of a DBS, which has a specific diameter of 5.9 mm. In order to estimate the total Hb content of the DBS, three derivatives of Hb were concurrently measured (oxyhemoglobin (OxyHb), methemoglobin (MetHb) and hemichrome). In vivo hemoglobin is constantly being oxidized to methemoglobin (ferric), with around 1% of hemoglobin being in this form at any time (20). It is crucial to estimate all these three Hb derivatives to determine the total Hb content of a DBS. During the method development, a direct comparison between different anticoagulants was also made since the anticoagulant choice during the preparation of the calibration model can influence the results. On the contrary, the spotted volume and the age of the DBS did not affect the predicted Hct value. The experiment showed a good agreement between predicted Hct and the actual Hct of the patients, measured via routine blood analysis. 95% of the predicted Hct values lied within 20% of the actual Hct. Eventually, the final method could determine the Hb content via a single wavelength reflectance measurement at 589 nm. The advantages were that it was nondestructive, and the preparation of the samples was not time consuming, with the possibility of the addition of an automated analysis of DBS.

For the estimation of Hct, the use of near infrared (NIR) spectroscopy was also investigated. Oostendorp et al. [83] focused on an NIR-spectrum approach for the determination of Hct from DBS. During the method development samples from 261 patients (male/female: 132/129; ages 0–95 years) with variable Hct values (0.15–0.60) were used to design the NIR model. The Hct results from the DBS analysis were compared with Hct estimated by the method of hemocytometry. These two methods showed a good correlation; the drying time of DBS, albumin concentration, age and sex of patients were non-significant covariates. The nondestructive character of the technique and the omission of sample preparation are the main benefits of this technique. Nevertheless, the small number of obtained samples during the method development, requires a further validation of this method.

#### 4.2.3. Hemoglobin Quantification Using UV-Visible Spectrometry

A destructive technique for the estimation of Hct was presented by Richardson et al. [84]. The principle of the developed method was based on the fact that Hb forms a complex with sodium lauryl sulphate (SLS). It has been demonstrated that the use of SLS, leads to the rapid conversion of OxyHb, deoxyhemoglobin, carboxyhemoglobin and MetHb to a sulfated derivative of Hb, which is stable for some hours at room temperature [85]. This SLS-Hb complex has an optimal absorbance range from 500 to 560 nm, and it can be easily measured by a UV-VIS spectrometer at 550 nm. In the first step of their approach, the authors performed a 6 mm punch on a DBS. After the extraction process, a commercially available agent that contains SLS (Sulfolyser) was added. The influence of various parameters (spotted volume of DBS, punch location, storage time and conditions) was also investigated. The stability of the samples was evaluated for a period of up to six months, with the samples being stored at 4 °C (<10% change). This very good long term-stability is one of the main advantages of this method, as well as the ability that SLS possesses of binding with all the forms of Hb. This enables the estimation of Hb in DBS samples that had been stored for a long-period, and in which the Hb has been converted to MetHb and hemichrome.

## 5. Recent Developments in Microsampling

**Figure 2:**
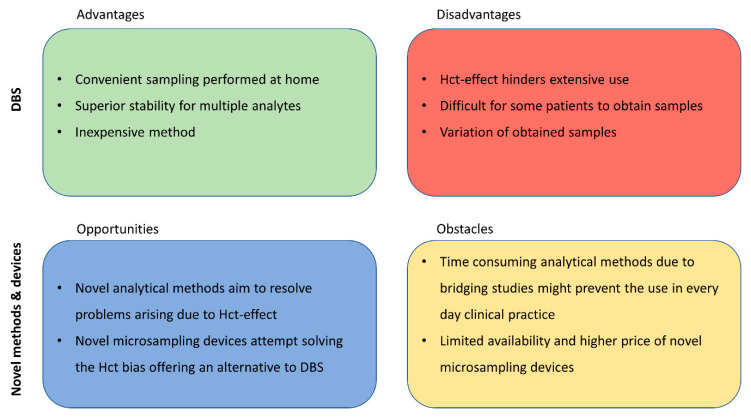
Current role of DBS compared to novel methods and devices.

To avoid the Hct effect, the DBS can be obtained by a volumetric approach, followed by the analysis of the whole DBS. This approach can be applied by either punching the whole DBS after volumetric application of the sample or volumetrically applying the blood on pre-punched discs. The disadvantage of the later is that it rules out the direct application of blood from the fingertip on the paper card. If patients directly apply a drop of blood from their fingertip on the paper card, they follow a non-volumetric approach (volume of blood is unknown). Over the past few years, a number of novel microsampling devices were developed, which try to overcome the Hct influence, but maintain the advantages of DBS [67,68,69]. Their main obstacles hindering their more extensive use are their limited availability and higher price (Figure 2).

### 5.1. Volumetric Blood Sample Collection

#### 5.1.1. hemaPEN

A novel microsampling device that has been developed by Trajan Scientific and Medical (Melbourne, Australia) is hemaPEN^®^ [86]. It has been used during the past few years for standard chromatographic and ligand binding assays for the quantification of biomarkers (immunoglobulins, cytokines, etc.). It is an advanced precision volumetric device which integrates a capillary based technology that allows the collection of four whole DBS with an accurate volume of 2.47 μL each, from a single whole blood source. When frequent sampling is required, the simplicity of use of hemaPEN is advantageous. The samples are better preserved, and they can be easily shipped without refrigeration by the patient. Protti et al. [87], utilized the hemaPEN microsampling technique, coupled with liquid chromatography with tandem mass spectrometry (LC-MS/MS) for the TDM of patients treated with drugs, acting on central nervous system (CNS). The method was validated based on the European Medicines Agency (EMA) and FDA guidelines. The effect the Hct might have on the extraction yield (demonstrated as % recovery) of the analytes and the matrix effect were assessed. The results of the recovery and matrix effect were considered independent of Hct, if the low Hct (0.3) and high Hct (0.7) samples collected using hemaPEN were within the limit of ±15% when compared to the recovery of the samples with medium Hct (0.5). Ultimately, it was demonstrated that the hemaPEN DBS performed without Hct-dependence, present an important step for the further implementation of this microsampling device. Deprez et al. [88] developed an LC-MS/MS method in order to determine caffeine and paraxanthine in DBS samples that were collected using hemaPEN. Similarly, this method was validated according to the guidelines on bioanalytical method validation by EMA and FDA. The concentrations of the analytes that were determined in hemaPEN DBS samples, demonstrated a low Hct-based bias of 6.9% (for caffeine) and 5.4% (for paraxanthine) when compared to venous whole blood samples (over the Hct range 0.20–0.50). Nevertheless, this effect was considered negligible, in the same comparison to sub-punched DBS, which showed a difference of over 25% over the same Hct range.

#### 5.1.2. Hemaxis

Hemaxis DB10 is an FDA class 1 certified whole blood collection device. It integrates a special microfluidic chip with a standard filter card and a protective case. Its main advantage is that it delivers an accurate and precise amount of blood without Hct bias. The repeatability in volume is comparable to a volumetric micropipette (e.g., VAMS) so there is no need for a sub-punch as the entire spot can be used for extraction [89]. The Hemaxis is described as a device with multiple benefits. It is a patient-centric microsampling device which can be delivered at patient’s home; the patient can complete the collection of blood or plasma by executing the finger prick. Zwart et al. [90], conducted their study for the TDM of the immunosuppressants tacrolimus and mycophenolic acid, by utilizing the Hemaxis DB10 device. Almost one fifth of the patients faced difficulties using the specific capillary device. Additionally, a 4.6% of patients encountered problems in clearly observing the device, due to impaired vision. However, the patients only received brief guidelines on how to obtain the samples themselves, which may justify their problems [91].

#### 5.1.3. VAMS

Neoteryx (USA) developed an alternative solution to the traditional DBS, named Mitra^®^. The principle of this device is based on VAMS. The Mitra^®^ microsampling device is an FDA listed class 1 exempt device and a CE-IVD self-certified in the UK and EU. A specific absorbent polymeric tip is attached to a plastic handler, allowing the easy collection of a pre-determined volume of blood (10, 20 or 30 μL) over a wide range of Hct values [92]. Even though it is a contemporary device, VAMS has shown very promising results in monitoring of various molecules (from small drugs to mAbs and peptides) [49]. De Kesel et al. [93], developed and validated a method, following the EMA and FDA guidelines for bioanalytical method validation in 2015, prior to the validation of the hemaPEN method. The concentrations of the analytes (caffeine and paraxanthine) using the VAMS microsampling technique were similarly compared to the matching DBS and whole blood sample, over a wide Hct range (0.21–0.50). It is worth noting that the concentrations obtained using the VAMS technique resulted in a consistently mean positive difference of 12.3% when compared to whole blood samples, independent of the Hct. This effect was attributed to different factors affecting the VAMS samples; it mainly had to do with the preparation of samples from incurred or spiked blood in combination with the decreased recovery of the analytes from the VAMS tips, in the case of high Hct.

Bloem et al. [63], performed a comparison between Mitra^®^ microsampler and Whatman^®^ filter paper assessing the effect Hct might have on the obtained samples. The dependency of the volume of blood and the Hct was not evident on samples obtained with the VAMS technique, characterized with a Hct range from 0.3 to 0.5. The researchers investigated the applicability of the Mitra^®^ microsampler for blood samples containing therapeutic mAbs. Blood was spiked with different mAbs and the corresponding recoveries were measured by ELISA, for both VAMS and Whatman^®^ filter paper samples. Both recoveries were close to 100%, but the Mitra^®^ microsampler samples showed less variation. The recovery of the samples was also evaluated in respect to storage time and conditions. Both types of obtained samples (Mitra^®^ microsampler and Whatman^®^ filter paper), were not influenced by the storage at room temperature or at 4 °C, for a period of one month. Similar results were obtained after storing both types of samples at 37 °C, for two days.

## 6. Conclusions

The growing importance of mAbs in the therapy of patients diagnosed with various inflammatory diseases is evident. The complex PK properties of mAbs, require careful TDM to reach a favorable therapeutic outcome. This can be achieved by frequent sampling, followed by analysis of the obtained samples. Microsampling could significantly assist the TDM of patients treated with mAbs. There have already been studies applying microsampling techniques for the determination of mAbs concentrations and the findings indicate that it is a convenient alternative to venipuncture. Despite the advantages of those techniques, some issues still hinder a more extensive application in clinical practice. Novel analytical methods and microsampling devices have been recently developed and they attempt to overcome those issues. Nevertheless, their implementation in clinical practice is still limited.

## Figures and Tables

**Table 1:** Recommended target trough serum concentrations associated with the favorable outcomes in the maintenance phase for common monoclonal antibodies in inflammatory diseases.

Antibody	Indication	Target trough Serum Concentration [μg/mL]	References
Adalimumab	IBD	≥7.5	[25,27]
	Pso	≥3.51	[34]
	RA	≥5	[23]
Certolizumab pegol	IBD	≥13	[25]
Infliximab	IBD	≥5	[25,27]
Golimumab	IBD	≥1	[25]

IBD, inflammatory bowel disease. Pso, Psoriasis. RA, rheumatoid arthritis.

**Table 2:** Summary of studies utilizing microsampling for the TDM of mAbs.

Antibody	Indication	Number of Patients	Microsampling Method	Lower Limit of Quantification (µg/mL)	Conversion of DBS Concentration into DBS-Serum Concentration	Stability	Extraction	Extraction Solution	Analytical Method	Reference
**Adalimumab**	RA, PsA, AS	n = 161: RA n = 96, PsA n = 31, AS n = 34	DBS-Whatman^®^ 903 Protein Saver Card	0.411	**DBS H0.42**: C_s_ = C_e_ × 1/(1 − 0.42) × V_e_/A (Hct) × (v_0_ + v_1_ × 0.42) **Correction Factor**: 1.19 = 1/(1 − 16.26%)	DBS cards spiked with anti-adalimumab at RT for up to 3 months	Overnight incubation (gently shaking) at RT	PBS/0.05% Tween 20/0.05% NaN_3_	ELISA	[53]
	IBD (UC and CD)	n = 33: CD n = 27, UC n = 6	Mitra^®^ microsampler-volumetric absorptive microsampling (VAMS)	0.6	A fixed **Hct (0.42)** was used for the conversion of DBS (VAMS) extracts to serum concentrations	-	Overnight vigorous shaking (≥17 h) on an orbital shaker	PBS/0.05% Tween 20/0.05% NaN_3_	ELISA	[54]
**Infliximab**	IBD (UC and CD)	n = 40: CD n = 29, UC n = 11	Mitra^®^ microsampler-volumetric absorptive microsampling (VAMS)	0.6	**DBS H-Hb**: serum fraction in DBS extract is calculated using Hct computed from haemoglobin in DBS extract	-	Overnight vigorous shaking (≥17 h) on an orbital shaker	PBS/0.05% Tween 20/0.05% NaN_3_	ELISA	[41]
**Golimumab**	UC	n = 10	DBS-Whatman^®^ 903 Protein Saver Card	0.2	**Correction factor 3.9**: combination of extraction recovery and the capillary blood/serum ratio for golimumab	DBS cards up to one month at RT, DBS extracts up to 3 months at −20 °C	1 h incubation, gentle shaking (300 rpm, RT) -> centrifugation at 14,000× *g* RCF (g), 5 min	SuperBlock^®^T20	ELISA	[55]
**Vedolizumab**	IBD (UC and CD)	n = 19: UC n = 9, CD n = 10	DBS-Whatman^®^ 903 Protein Saver Card	2	**[VDZ]_DBS_** = [VDZ]_serum_ × 0.435 + 0.995 (R^2^ = 0.956)	DBS cards up to 1 month at RT/extracts at −20 °C for 3.5 months-no influence on VDZ recovery	1 h incubation, gentle shaking (300 rpm, RT) -> centrifugation at 14,000× *g* for 5 min	SuperBlock^®^T20	ELISA	[56]

RA, rheumatoid arthritis. PsA, psoriatic arthritis. AS, ankylosing spondylitis. IBD, inflammatory bowel disease. UC, ulcerative colitis. CD, Crohn’s disease. DBS, dried blood spot; VAMS, volumetric absorptive microsampling. C_s_, serum concentration. C_e_, concentration in DBS eluate. V_e_, volume of the eluate. A(Hct), area of the DBS. v_0_, v_1_ = parameters determined by Hct dependent spreading of blood on filter paper. RT, room temperature. PBS, Phosphate buffer saline. NaN_3_, Sodium azide. rpm, revolutions per minute. RCF, relative centrifugal force. [VDZ]_DBS_, Vedolizumab concentration in DBS. [VDZ]_serum_, Vedolizumab concentration in serum. ELISA, enzyme-linked immunosorbent assay.
